# Evaluating the effect of targeted strategies as control tools for hypervirulent meningococcal C outbreaks: a case study from Tuscany, Italy, 2015 to 2016

**DOI:** 10.2807/1560-7917.ES.2023.28.19.2200650

**Published:** 2023-05-11

**Authors:** Giorgio Guzzetta, Marco Ajelli, Alessandro Miglietta, Cecilia Fazio, Arianna Neri, Stefano Merler, Giovanni Rezza, Paola Stefanelli

**Affiliations:** 1 Center for Health Emergencies, Bruno Kessler Foundation, Trento, Italy; 2 Laboratory for Computational Epidemiology and Public Health, Department of Epidemiology and Biostatistics, Indiana University School of Public Health, Bloomington, Indiana, United States; 3 Units of Epidemiology and Preventive Medicine, Central Tuscany Health Authority, Florence, Italy; 4 Regional Health Agency of Tuscany, Epidemiologic Observatory, Florence , Italy; 5 Department of Infectious Diseases, Istituto Superiore di Sanità, Rome, Italy; 6 Health Prevention Directorate, Ministry of Health, Rome, Italy

**Keywords:** Serogroup C meningococcus, outbreak control, vaccination, contact tracing, agent-based model

## Abstract

**Background:**

Meningococcus (*Neisseria meningitidis*) is the causative bacteria of invasive meningococcal disease (IMD), a major cause of meningitis and sepsis. In 2015–16, an outbreak caused by serogroup C meningococci (MenC), belonging to the hyperinvasive strain ST-11(cc-11), resulted in 62 IMD cases in the region of Tuscany, Italy.

**Aim:**

We aimed to estimate the key outbreak parameters and assess the impact of interventions used in the outbreak response.

**Methods:**

We developed a susceptible-carrier-susceptible individual-based model of MenC transmission, accounting for transmission in households, schools, discos/clubs and the general community, which was informed by detailed data on the 2015–16 outbreak (derived from epidemiological investigations) and on the implemented control measures.

**Results:**

The outbreak reproduction number (R_e_) was 1.35 (95% prediction interval: 1.13–1.47) and the IMD probability was 4.6 for every 1,000 new MenC carriage episodes (95% confidence interval: 1.8–12.2). The interventions, i.e. chemoprophylaxis and vaccination of close contacts of IMD cases as well as age-targeted vaccination, were effective in reducing R_e_ and ending the outbreak. Case-based interventions (including ring vaccination) alone would have been insufficient to achieve outbreak control. The definition of age groups to prioritise vaccination had a critical impact on the effectiveness and efficiency of control measures.

**Conclusions:**

Our findings suggest that there are no effective alternatives to widespread reactive vaccination during outbreaks of highly transmissible MenC strains. Age-targeted campaigns can increase the effectiveness of vaccination campaigns. These results can be instrumental to define effective guidelines for the control of future meningococcal outbreaks caused by hypervirulent strains.

**Key public health message**

**What did you want to address in this study?**

Outbreaks of certain serogroup C meningococcal strains, which cause severe and often lethal forms of meningitis and sepsis, are an important and current public health threat. Using data from an outbreak in Tuscany, Italy, during 2015–16, we ran digital simulations reproducing the observed data and then assessed the impact of different outbreak control strategies that could have been implemented.

**What have we learnt from this study?**

The combination of antibiotic treatment and immunisation of close contacts, and vaccination of the general population with a priority for ages 11–20 years, was an appropriate strategy to contain and control the outbreak. Expanding the main target age group for vaccination to 25 years of age would result in better outbreak control, and decrease the number of cases faster.

**What are the implications of your findings for public health?**

Our study shows that widespread reactive vaccination campaigns should be the preferred strategy during outbreaks of highly transmissible meningococcal strains. Contact tracing prevents cases, but without significantly reducing bacterial circulation. Moreover, tracing contacts of contacts (known as ring vaccination) would have alone been insufficient to bring the outbreak under control, and inefficient when in combination with effective interventions like widespread vaccination.

## Introduction

*Neisseria meningitidis*, also known as meningococcus, may cause sporadic cases, clusters or large outbreaks of invasive meningococcal disease (IMD). Asymptomatic carriage of these bacteria is relatively common, but infection can occasionally result in IMD, which is characterised by a high lethality and a high probability of permanent sequelae. Six serogroups of *N. meningitidis* (A, B, C, W, X and Y) are most frequently responsible for life-threatening disease, and pathogenic strains belong to few genetically defined clonal complexes (cc) [1-3]. Some hypervirulent isolates (sequence type (ST)-11(cc-11) strain) belonging to serogroup C (MenC) have been associated with outbreaks of severe disease [4]. These strains are more likely to be transmitted, present a shorter duration of carriage [5,6] and involve a higher risk of invasive disease compared with other meningococcal strains, with most cases occurring within a few days after infection [7,8]. 

Chemoprophylaxis of close contacts is effective in minimising the risk of secondary cases [9] but may not be sufficient to control bacterial circulation. Thus, reactive vaccination can be an important option for outbreak control. The extent of the population that should be vaccinated to control an outbreak may depend on the setting where the outbreak occurs (i.e. organisation vs community-based outbreaks vs mixed outbreaks [10]) and on strain virulence and population susceptibility (i.e. coverage of routine vaccination schedules). Age-targeted or even mass vaccination is an option that should be considered for community-based outbreak control [11,12]. Such strategies have been successfully applied to control serogroup A epidemics in central Africa [13], but little information is available on outbreak control in high-income countries, and specifically for hyperinvasive MenC strains. 

During 2015–16 in Tuscany, Italy, a large MenC outbreak affected the gay and bisexual community totalling 62 IMD cases, of which 13 were fatal [14]. The first IMD case in the outbreak occurred on 6 January 2015 and was followed by 11 more cases in the region by March 2015, prompting the launch of a reactive age-targeted vaccination campaign on 30 March [15]. This outbreak was later found to be caused by a clonal expansion of a unique hyperinvasive and hypervirulent MenC ST-11(cc-11) [15], which was amplified by overcrowded attendance to discos and clubs [14]. Here, we calibrated a computational model to reproduce data from this outbreak and assessed the impact of contact tracing and vaccination strategies on the reduction of IMD incidence by running the same outbreak under different implementation options (e.g. ring vaccination, age-targeted and mass vaccination).

## Methods

### Modelling transmission of serotype C meningococcus

We developed an individual-based model of MenC transmission within households, schools, discos/dancing clubs and in the general population [16-19]. This choice was based on results from epidemiological investigations of the 2015–16 outbreak in Tuscany, Italy, which suggested that 53% of the 62 IMD cases (n = 33) were likely acquired in discos and dancing clubs, 19% (n = 12) via household contacts and 13% (n = 8) in schools [14]. Available data on the distribution of discos/clubs admittance capacity [20] and on age-specific time-use data [21,22] were used to model disco/club attendance. We adopted a susceptible-carrier-susceptible structure, where all new carriers have a probability to develop IMD shortly after meningococcal acquisition, and a probability of death by IMD. 

**Figure 1:**
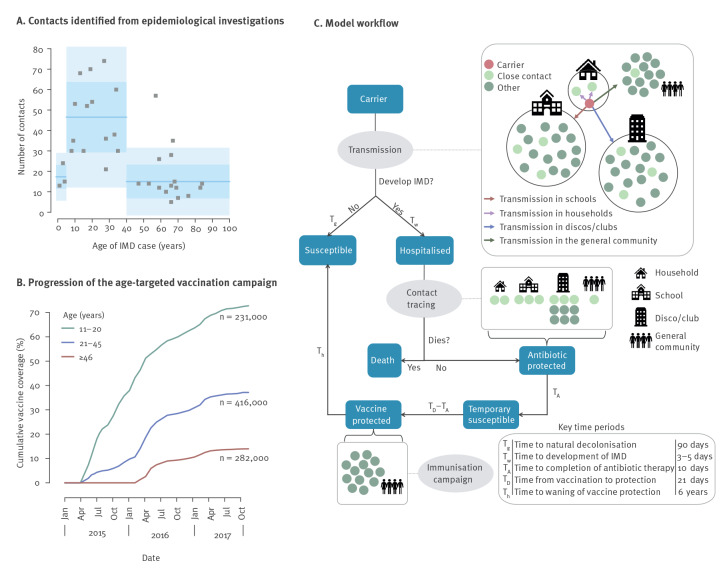
Individual-based model for meningococcus C transmission during an outbreak in Tuscany, Italy, 2015–2016

Transmission in households, schools, discos/clubs and in the community is regulated by four setting-specific transmission rates. Each individual was assigned a number of close contacts from contact tracing data in the considered outbreak (Figure 1A) distributed across schools, discos/clubs and in the population at random depending on the individual’s age, according to data from the POLYMOD contact surveys for Italy [23]. In addition, all household members of an individual were considered close contacts. Close contacts were assumed to have an increased relative risk (RR) of carriage compared with other contacts in the same setting (estimated at ca 2.7-fold [24]). The model was implemented in C using GNU scientific libraries version 2.6 (https://www.gnu.org/software/gsl).

IMD: invasive meningococcal disease.

A. Number of traced close contacts, by age of IMD case, obtained from epidemiological investigations conducted during the outbreak. Close contacts were assigned to each individual in the model by sampling Gaussian distributions, with age-specific parameters computed from outbreak data [14] (grey squares). Blue lines represent the mean, while bands represent one and two standard deviations around the mean for each age group.

B. Age group-specific cumulative coverage of the actually implemented reactive immunisation campaign over time. Numbers reported at the end of each curve represent the total number of vaccines administered by the end of 2017 for the corresponding age group.

C. Schematic workflow of the model summarising the natural history of infection, the modelled transmission dynamics and the implemented interventions.

### Control interventions

To reproduce the outbreak dynamics, we modelled the interventions that were actually implemented, assuming that all individuals who developed IMD and survived received antibiotic chemoprophylaxis and vaccination on the same day. Chemoprophylaxis removes carriage and confers complete protection from the infection for the whole duration of therapy; after this period, individuals revert to full susceptibility to carriage. Vaccine protection was assumed to develop for all individuals about 3 weeks after immunisation [25,26], and to wane after an exponentially distributed time (mean: 6 years) [25]. We assumed that all close contacts of an IMD case were traced, in addition to a fraction of individuals who attended the same discos as the ones (if any) attended by the index case in the 2 weeks preceding symptom onset. Traced contacts were assumed to receive chemoprophylaxis and vaccination on the same day of disease development. 

Reactive age-targeted vaccination campaigns were implemented by administering the vaccine to random individuals with probability proportional to daily age group-specific coverage data (ages 11–20, 21–45 and > 45 years), starting from 30 March 2015 (Figure 1B).

The model was used to assess the effect of alternative combinations of interventions, considering the administration of antibiotic chemoprophylaxis, *A*, and vaccines, *V*, to different groups of individuals (close contacts, *c*, contacts of contacts *cc*, selected age targets, *a*, or the generic population, *m*). The interventions that were actually implemented to contain the outbreak were chemoprophylaxis and vaccination to close contacts of IMD cases (including a fraction of attendees to the same disco, if any), and reactive vaccination with age-specific coverage: according to the above notation, this is represented as A_c_ + V_c_ + V_a_ (baseline scenario). A scenario where vaccination is administered regardless of the individual’s age, implemented so that the same total number of vaccines over time is given across the population rather than in the selected age groups, was denoted as V_m_ (mass vaccination). 

Figure 1C shows a schematic workflow of the model, including the natural history of infection and implemented interventions. Please see Supplementary Material S1 for a full description of the model, including initialisation and parameter values.

### Model calibration and sensitivity analyses

Free model parameters were the four transmission rates (in households, schools, discos/clubs and in the community) and the probability of IMD given a new MenC carriage. We fit the total number of IMD cases identified by epidemiological investigations as household-related, school-related, disco-related, or in the community (i.e. without a known epidemiological link to other IMD cases) in 2015–16 [14], using a Markov Chain Monte Carlo approach to explore the likelihood of observing the setting- and year-specific number of cases given the parameters of the model (see Supplementary Material S2 for details on calibration). After calibration, for each intervention scenario, we computed the effective reproduction number (R_e_) by counting the number of carriers that were infected by each carrier throughout their infectious period.

We assessed the robustness of our results by running sensitivity analyses with respect to several alternative modelling choices, including the proportions of contacts traced in discos/clubs, the RR of carriage among close contacts, the delay between vaccination and the mounting of protective immunity, and the algorithm for selecting attendants to a disco (see Supplementary Material S4 for details on sensitivity analysis implementation and results).

## Results

**Figure 2:**
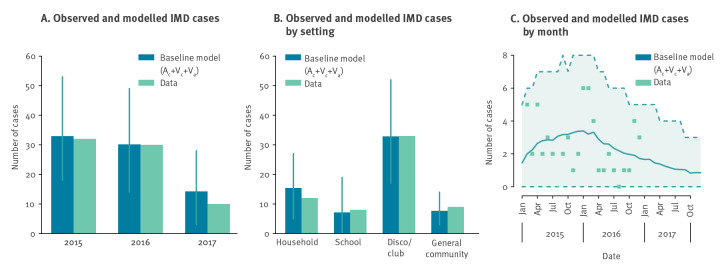
Model calibration and validation of a model of a meningococcus C outbreak, Tuscany, Italy, 2015–2016

The calibrated model of MenC transmission for the baseline scenario was able to reproduce the number of yearly IMD cases and the distribution of the source of exposure (Figure 2A-B) [14]. The model correctly predicted the observed decline of the outbreak in 2017, with an average estimate of 14.2 IMD cases (95% prediction interval (PI): 3–28) compared with the 10 cases recorded in the region (data point not used for calibration). In addition, the model reproduced the stochastic variability of observed cases over time well (Figure 2C).

A_c_: antibiotic chemoprophylaxis administered to close contacts; IMD: invasive meningococcal disease; PI: prediction interval; V_a_: age-targeted vaccination campaign; V_c_: vaccination administered to close contacts.

A. Observed number of IMD cases by year over the period 2015–17 and corresponding average number of IMD cases as estimated by the model (vertical bars represent 95% PI over 1,000 simulations). Data for 2017 were not used during calibration and are reported as a model validation.

B. Observed number of IMD cases by setting of acquisition (2015–16) and corresponding average number of IMD cases as estimated by the model (vertical bars represent 95% PI over  1,000 simulations). Observed data are derived from published cluster investigations [14]. We aggregated those cases for which the source of exposure was not determined or was in a setting different from households, schools and discos/clubs into transmission in the general community.

C. Observed monthly number of IMD cases (green points) and corresponding average monthly number of IMD cases (blue solid line) as estimated by the model (blue dashed lines and shaded area represent 95% PI over 1,000 simulations). Monthly disaggregated data for 2017 were not available.

### Effect of the implemented interventions

**Figure 3:**
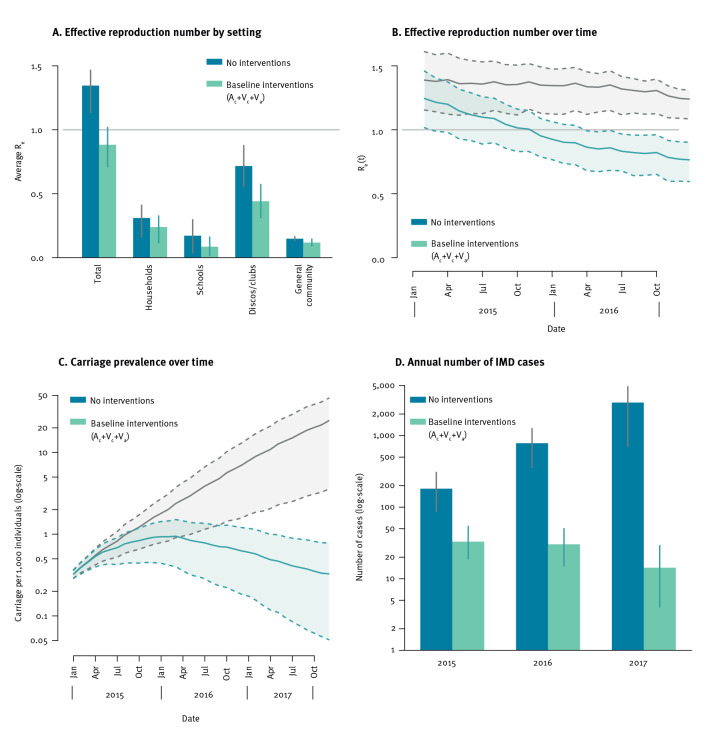
Model estimates for the effect of the implemented interventions during a meningococcus C outbreak, Tuscany, Italy, 2015–2016

We estimated the outbreak’s effective reproduction number (R_e_) in the absence of interventions at 1.35 (95% PI: 1.13–1.47) (Figure 3A). The implemented interventions (baseline scenario) were able to force R_e_ below the epidemic threshold, at 0.88 (95% PI: 0.71–1.02), reducing most notably the transmissibility contribution of discos/clubs (by 0.29 units) and schools (by 0.08 units) (Figure 3A). The larger effect obtained in these settings may be attributed to the prioritisation of the immunisation campaigns to the age group 11–20 years, and to contact tracing activities targeted to individuals attending the same discos/clubs of IMD cases. The model suggests that R_e_ fell below the epidemic threshold around December 2015 and reached a value of 0.77 (95% PI: 0.60–0.91) by December 2016 (Figure 3B). The probability of IMD given a new MenC carriage was estimated at 4.6 IMD cases per 1,000 new carriage episodes (95% confidence interval (CI): 1.8–12.2) and the overall prevalence of MenC carriage over time was estimated to reach a maximum of 0.93 (95% PI: 0.36–1.52) carriers per 1,000 individuals in March 2016 (Figure 3C). In the absence of interventions, both the MenC carriage prevalence and the number of IMD cases would have increased exponentially (Figure 3C-D). The implemented interventions (A_c_ + V_c_ + V_a_) could successfully contain MenC outbreaks characterised by values of the R_e_ up to about 1.5 (see Supplementary Material S3 for additional simulations with different values of R_e_).

A_c_: antibiotic chemoprophylaxis administered to close contacts; IMD: invasive meningococcal disease; V_a_: age-targeted vaccination campaign; V_c_: vaccination administered to close contacts.

A. Effective reproduction number (average over the period January–June 2016) and by setting of transmission, with and without interventions. Vertical bars represent 95% PI over 1,000 simulations.

B. Effective reproduction number over time, with and without interventions (solid lines). Dashed lines and shaded areas represent 95% PI over 1,000 simulations.

C. Average carriage prevalence over time, with and without interventions (solid lines, y-axis on a log-scale). Dashed lines and shaded areas represent 95% PI over 1,000 simulations.

D. Average yearly number of IMD cases (y-axis on a log-scale), with and without interventions. Vertical bars represent 95% PI over 1,000 simulations.

### Effect of alternative intervention strategies

**Figure 4:**
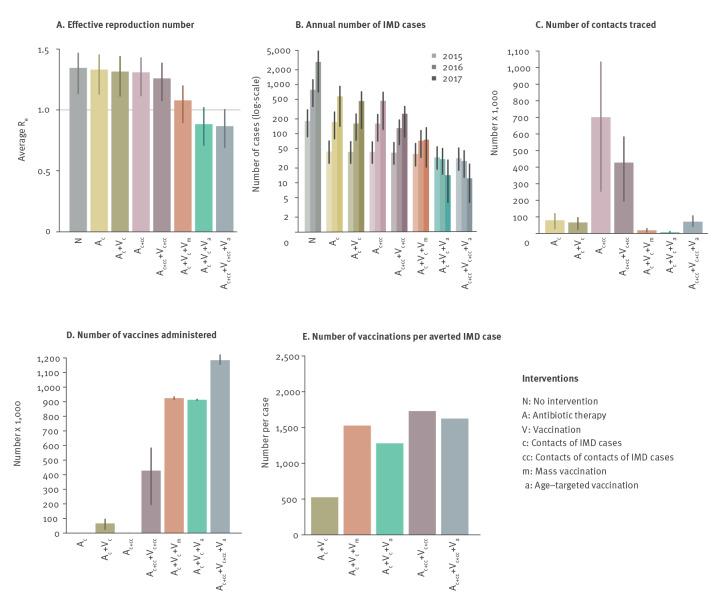
Model estimates for the effect of alternative interventions during a meningococcus C outbreak, Tuscany, Italy, 2015–2016

We found that control strategies not including an age-targeted (V_a_) or a mass (V_m_) immunisation campaign would be insufficient to contain the outbreak (Figure 4A), resulting in rapidly growing numbers of IMD cases over time (Figure 4B). The implemented interventions (A_c_ + V_c_ + V_a_) were successful in bringing the outbreak under control while at the same time minimising the number of contacts to be traced (Figure 4C). The model estimated an average of 6,300 contacts traced over the period 2015–16 (95% PI: 3,500–9,700), which is in line with the ca 7,000 contacts traced during the actual outbreak [26]. The immunisation campaign required a large number of vaccines to be administered (estimated average: 913,000; 95% PI: 910,000–917,000, in line with the 929,000 immunisations actually performed) (Figure 4D). Among strategies that do not consider a widespread immunisation campaign, the best performance in terms of IMD case reductions would be obtained by a scenario (A_c + cc_ + V_c + cc_, ring vaccination) which includes the administration of antibiotics and vaccines to both close contacts of IMD cases (primary contacts), and to close contacts of the primary contacts (secondary contacts). With this strategy, the total number of IMD cases expected over the period 2015–17 would be 5.5-fold higher than observed. In addition, the lower number of vaccine doses administered (ca 427,000, 95% PI: 196,000–582,000) would be offset by the need to trace individually and treat with antibiotics a large fraction of the total population (ca 15%) and by the risk of inducing antibiotic resistance when administering antibiotics at this scale [27]. If the additional tracing of secondary contacts was combined with the implemented age-targeted vaccination (scenario A_c + cc_ + V_c + cc_ + V_a_), the number of IMD cases would be further reduced by 7.6% with respect to what has been observed, but the effort required for tracing contacts would be ca 9.3 times higher (ca 70,700 contacts) than the baseline. For any scenarios including the use of vaccines, we computed the average number of immunisations required to avert an IMD case with respect to a corresponding scenario where only chemoprophylaxis is administered to traced contacts. The strategy implemented within the current outbreak was more efficient than all other scenarios (Figure 4E), except for one where only primary contacts are immunised (A_c_ + V_c_, which, however, has a very poor performance in terms of outbreak control).

A_c_: antibiotic chemoprophylaxis administered to close contacts; A_c+cc_: antibiotic chemoprophylaxis administered to close contacts and contacts of contacts; IMD: invasive meningococcal disease; N: no intervention; V_a_: age-targeted vaccination campaign; V_c_: vaccination administered to close contacts; V_c+cc_: vaccination administered to close contacts and contacts of contacts; V_m_: mass vaccination without age-prioritisation.

A. Effective reproduction numbers for different intervention strategies (average over the period January–June 2016). Vertical bars represent 95% PI over 1,000 simulations.

B. Average yearly number of IMD cases (y-axis on a log-scale) in 2015, 2016 and 2017. Vertical bars represent 95% PI over 1,000 simulations.

C. Average total number of contacts traced over the period 2015–17 (in thousands). Vertical bars represent 95% PI over 1,000 simulations.

D. Average total number of vaccines administered over the period 2015–17 (in thousands). Vertical bars represent 95% PI over 1,000 simulations.

E. Average number of vaccinations per averted IMD case. For each scenario, averted cases were computed with respect to the corresponding baseline in which no vaccination is performed but the same type of contacts are traced.

The baseline interventions were A_c_ + V_c_ + V_a_.

### Age-prioritisation of the vaccination campaign

**Figure 5:**
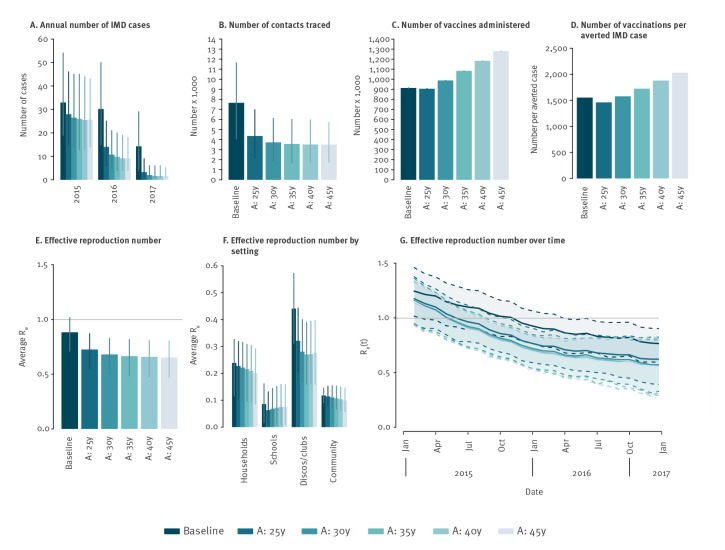
Model estimates for the effect of age prioritisation in vaccination campaigns during a meningococcus C outbreak, Tuscany, Italy, 2015–2016

In the considered outbreak, the age-targeted vaccination campaign achieved a 51% coverage within the age group 11–20 years 1 year after the start of the campaign; coverage in the age group 21–45 years rose more slowly, to 18% after the first year (Figure 1B). We tested the effect of extending the rapid coverage campaign to progressively older ages (Figure 5). We found that including young adults up to 25 years of age would have resulted in a 58.2% (95% PI: 56.3–61.5) reduction in the total number of IMD cases over the period 2015–17 (Figure 5A). The extra vaccine doses necessary for the increased coverage among the group aged 21–25 years would be compensated by the reduction in the number of traced and vaccinated contacts (Figure 5B and 5C). Therefore, the number of vaccines per averted cases would decrease slightly from ca 1,550 in the baseline scenario to ca 1,460 (Figure 5D). Furthermore, extending the rapid coverage to older individuals (ages 30–45 years who were generally exposed to infectious carriers at a lower frequency than younger cohorts), would only result in a marginal further reduction in the number of cases while incurring progressively higher intervention costs (in terms of vaccine doses) for public health. Extending the rapid coverage to individuals up to 25 years of age would reduce the average R_e_ with respect to the baseline (Figure 5E), leading to a faster decline after the beginning of the campaign. Indeed, in this scenario, R_e_ was expected to fall below the epidemic threshold in June 2015 (Figure 5G), i.e. only 3 months after the start of the vaccination campaign and 6 months earlier than the estimate for the baseline scenario. Much of the decline is because of further reductions in transmission within discos/clubs (Figure 5F). In this scenario, the peak of MenC carriage is expected to be 0.67 (95% PI: 0.34–0.96) carriers per 1,000, i.e. a reduction of 28% from the baseline, in September 2015.

IMD: invasive meningococcal disease.

A. Average yearly number of IMD cases. Vertical bars represent 95% PI over 1,000 simulations.

B. Average total number of contacts traced over the period 2015–17 (in thousands). Vertical bars represent 95% PI over 1,000 simulations.

C. Average total number of vaccines administered over the period 2015–17 (in thousands). Vertical bars represent 95% PI over 1,000 simulations.

D. Average number of vaccinations per averted IMD case. Averted cases are computed with respect to a scenario where only chemoprophylaxis of contacts is performed.

E. Effective reproduction number (average over the period January–June 2016). Vertical bars represent 95% PI over 1,000 simulations.

F. Effective reproduction number by setting of transmission (average over the period January–June 2016). Vertical bars represent 95% PI over 1,000 simulations.

G. Effective reproduction number over time (solid lines). Dashed lines represent 95% PI over 1,000 simulations.

Prioritisation of the vaccine is assumed for individuals older than 11 years, and up to a given age (A) (baseline: 20 years).

## Discussion

Although meningococcal incidence is declining in Europe and the United States (US), thanks to the introduction of effective vaccines [3], outbreaks of hyperinvasive and hypervirulent meningococcal strains are still a significant public health problem. The major MenC ST-11(cc11) outbreak we examined here disproportionately affected the gay and bisexual community and their contacts, and about half of cases occurred in clusters of transmission that were linked to gay venues, pride parties or homosexual contacts [14]. Other MenC outbreaks among men who have sex with men have occurred in several parts of the world [28,29] and, at the time of writing, a large outbreak involving the gay and bisexual community is ongoing in Florida, US, with 86 cases registered between 1 December 2021 and 11 April 2023 [30,31].

We analysed the 2015–16 Tuscany outbreak by means of an individual-based model of the infection transmission process, considering the socio-demographic structure of the population, transmission within households, schools, discos/clubs and in the general community, as well as the implemented control interventions. We estimated the R_e_ in the absence of interventions as ca 1.35 (95% PI: 1.13–1.47), which confirms the high transmissibility of the outbreak strain. For comparison, the R_e_ for non-hyperinvasive serogroup C meningococci had been estimated at 1.36 in a fully susceptible population [32]. However, in Tuscany, over 80% of individuals aged 2 to 20 years had been vaccinated before the beginning of the outbreak (both at birth and during catch-up campaigns) [33].

The strategy implemented to curtail the outbreak consisted in offering antibiotic therapy and vaccination to close contacts of IMD cases (traced via epidemiological questionnaires) and to customers of the same discos/clubs attended by cases (if any) in the previous 2 weeks. Furthermore, a population-wide vaccination campaign was implemented, prioritising individuals aged 11–20 years. This age group had been the target of a catch-up vaccination program in 2007 after the introduction in 2006 of routine vaccination at age 13–15 months [34]. We found that these interventions were sufficient to end the outbreak, that they could be effective for containing MenC outbreaks with R_e_ up to ca 1.5 and that they did so efficiently with respect to possible alternative strategies. In particular, we demonstrated that, because of the mostly asymptomatic circulation of meningococci, any strategy targeting only cases and their primary or even secondary contacts (i.e. ring vaccination) would have been insufficient to curb the outbreak. We also showed that expanding the implemented strategy with the adoption of ring vaccination would not result in a significant reduction of cases and would require a large additional effort. The poor effectiveness of ring vaccination around IMD cases depends mainly on the low rates of MenC carriage (estimated at less than 1 per 1,000 in the general population), even when accounting for an almost threefold RR in close contacts of carriers compared with the general population [24]. Most importantly, we estimated that if the group aged 21–25 years were included in the prioritisation of the vaccination campaign, a much more rapid reduction in R_e_ could be expected, resulting in less than half of the total IMD cases over the period 2015–17. This result depends on the combination of the high transmissibility of meningococci in discos/clubs and on the distribution of the proportion of individuals attending discos/clubs, which was shown to peak precisely in the age group 21–25 [20]. Indeed, the highest incidence of invasive disease was observed among this age group [14]; however, despite performing an ad hoc carriage prevalence study during the outbreak, the age-specific carriage prevalence could not be determined because of the low numbers of positive individuals identified [34]. The importance of identifying the appropriate age groups for implementing age-targeted vaccination is supported by the much lower effectiveness estimated for mass vaccination with respect to age-targeted vaccination.

The model reproduced the observed number of IMD cases in the study period 2015–16, their distribution by setting of acquisition and the stochastic variability of cases over time. The model also predicted with good accuracy the decrease in the number of IMD cases for year 2017 that was afforded by control interventions. Model estimates for the probability of IMD given a new MenC carriage (4.6 cases per 1,000 new carriage episodes, 95% CI: 1.8–12.2) were in line with previous literature estimates (ranging between 0.8 and 11.1 per 1,000 [35]). 

Our study had some limitations. Firstly, the model could not account for the spatial heterogeneity of transmission; we considered the population of Tuscany provinces affected by the outbreak to be closed and spatially well-mixed. We mitigated this assumption for disco/club transmission by assigning each individual in the model a limited number of venues that could be attended, in order to represent the lower likelihood of attending venues that are farther from an individual’s residence. Secondly, we could not consider the possibility that vaccine uptake during the campaign might have been locally higher in communities with more IMD cases. Thirdly, although the outbreak disproportionally affected the gay and bisexual community, we did not explicitly model potential differences in contact rates between this community and the rest of the population because of the absence of data. Fourthly, the grouping of cases with no epidemiological links in the ‘general community’ category of transmission might be incorrect if epidemiological links to schools or discos/clubs was not identified by epidemiological investigations. However, this bias is expected to be low since the proportion of unlinked cases was relatively small (15%). Finally, there was a high uncertainty on some parameters and modelling choices, including the proportions of contacts traced in discos/clubs, the RR of carriage among close contacts, the delay between vaccination and the mounting of an effective immunity, and choices on who is selected to attend to discos/clubs in the model. Nonetheless, a sensitivity analysis on each of these uncertainties was conducted (see Supplementary Material S4 for the sensitivity analyses) and showed the robustness of our conclusions. In addition to limitations listed above, we note that we choose not to model an alternative intervention scenario where a targeted vaccination campaign was implemented earlier. During this outbreak, reactive vaccination started less than 3 months after the first recorded case and a pronounced anticipation of implementation would have not been realistically feasible.

## Conclusion

We used data from a 2-year-long hyperinvasive MenC outbreak to calibrate a highly detailed transmission model, which allowed the evaluation of the most effective containment strategies. Our model results suggest that there are no alternatives to widespread reactive vaccination to control outbreaks of highly transmissible serogroup C meningococcal strains, and that a correct prioritisation of age groups for vaccination can increase the effectiveness and efficiency of immunisation campaigns. Although the modelling exercise was tailored to reflect the Italian socio-demographic situation, as well as to reconstruct the main epidemiological features of the 2015–16 MenC ST-11(cc11) outbreak in Tuscany, the proposed framework highlighted general features of serogroup C meningococcal transmission and control and could be instrumental to define effective guidelines for the containment of current and future outbreaks caused by hypervirulent strains.

## Supplementary Data

603000 Supplement
