# Supplementary material for: Evaluating the effect of targeted strategies as control tools for hypervirulent meningococcal C outbreaks: a case study from Tuscany, Italy, 2015 to 2016
Source: Euro Surveill. 2023 May 11;28(19):2200650. doi: 10.2807/1560-7917.ES.2023.28.19.2200650 (PMC10176827; doi:10.2807/1560-7917.ES.2023.28.19.2200650)
Supplement: Supplement [file 22-00650_MERLER_SUPPLEMENT.pdf]

Supplementary Material

This supplementary material is hosted by Eurosurveillance as supporting information alongside the article **Evaluating the effect of targeted strategies as control tools for hypervirulent meningococcal C outbreaks: a case study from Tuscany, Italy, 2015 to 2016** on behalf of the authors who remain responsible for the accuracy and appropriateness of the content. The same standards for ethics, copyright, attributions and permissions as for the article apply. Supplements are not edited by Eurosurveillance and the journal is not responsible for the maintenance of any links or email addresses provided therein.

Table of Contents

Supplementary Material S1 - The model of hyperinvasive MenC transmission 2
Socio-demographic model of the population of Tuscany 2
Close contacts 4
Model initialization 5
Transmission dynamics 5
Supplementary Material S2 – Model calibration 7
Supplementary Material S3 - Scenario analysis 8
Supplementary Material S4 - Sensitivity analyses 10
Delay between vaccination and the mounting of protective immunity, T_D 10
Relative risk of carriage among close contacts, ϕ 11
Fraction of traced disco/club attendees, z 12
Modeling of disco/club attendees 14
Summary of sensitivity analysis results 15
Supplementary References 16

Supplementary Material S1 - The model of hyperinvasive MenC transmission

Socio-demographic model of the population of Tuscany

We developed a synthetic population where the 2.9 million residents of the Tuscan provinces involved by the outbreak are co-located in synthetic households and schools according to the observed sociodemographic statistics. The procedure used to generate synthetic households and schools is reported in detail in Fumanelli et al. [S1]. A comparison between model output and key demographic statistics of the Italian population is shown in Supplementary Figure S1.

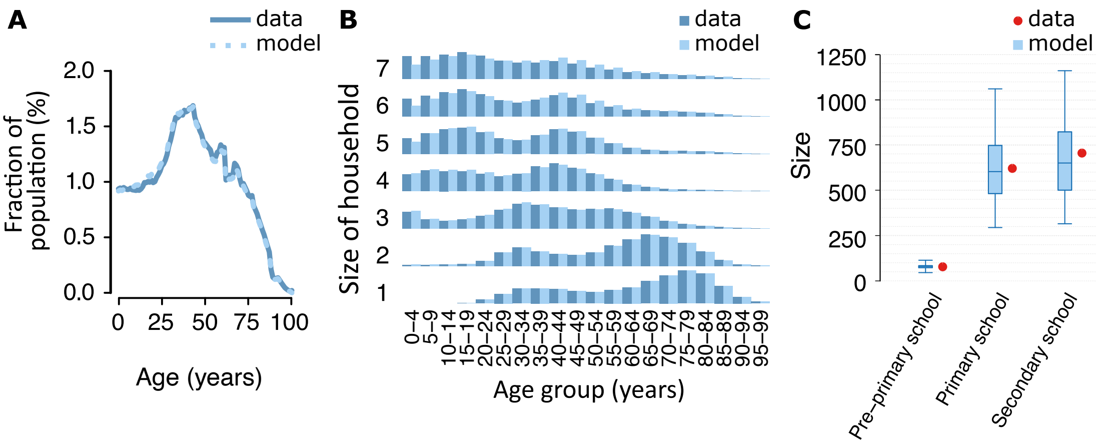

Supplementary Figure S1. Comparison between population model and data. A. Age structure of the population. B. Age distribution of individuals living in households of different sizes. C. Size of schools by level of education.

Due to the remarkable proportion (~53%) of IMD cases linked to transmission in discos and dancing clubs during the 2015-2016 MenC outbreak in Tuscany, we consider in the model 270 venues of this kind in the area, with maximum admittance sampled uniformly between 75 and 750 persons (median size 412.5, total admittance about 111,000), according to available data [S2]. At the beginning of each simulation, each individual was assigned a list of possible venues that she/he can possibly visit. The number of favorite discos/clubs was sampled from a Poisson distribution (mean 1.94), which resulted as the best fit to data from epidemiological investigations [S3] (see Supplementary Figure S2). Favorite discos/clubs were assigned to each individual with probability proportional to a venue's maximum admittance. Discos/clubs were assumed to be empty for six days a week and customers were allocated only one day a week (see Supplementary Table S1) [S4].

Day	Sample size	Probability (%)	Lower 95% CI	Upper 95% CI
Saturday	5853	7.31	6.66	8.01
Sunday	5728	1.34	1.06	1.68
Workday	6425	1.01	0.78	1.29

Supplementary Table S1. Probability of attending a disco/club by any person of age between 16 and 40 years old, on Saturday, Sunday and workday [S4]. Sample size is the number of questionnaires compiled by study participants of age between 16 and 40 years old.

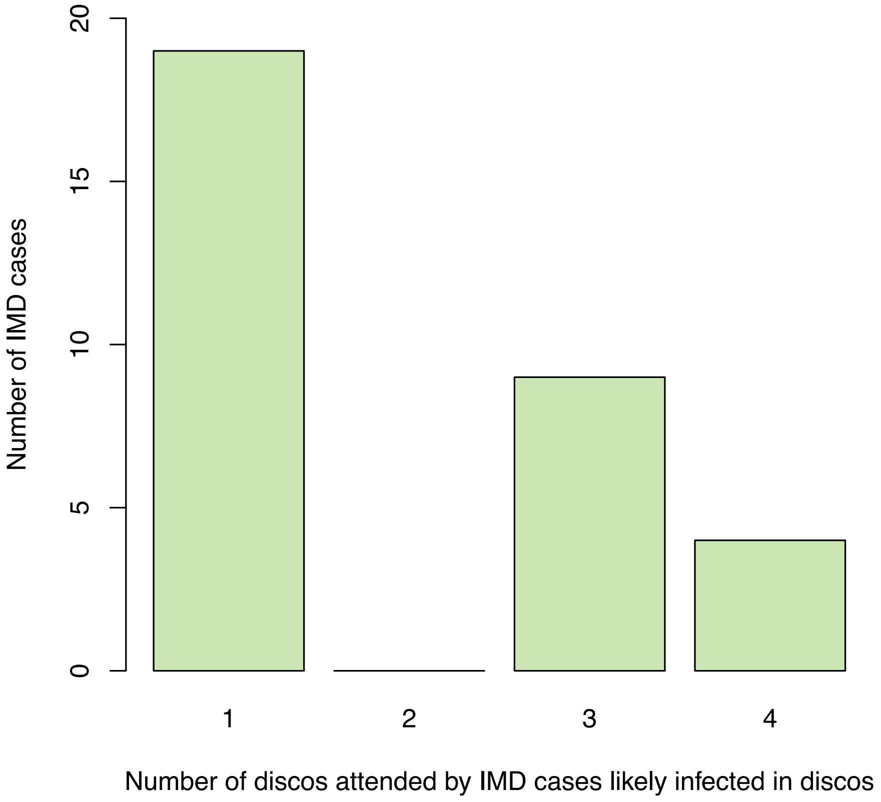

Supplementary Figure S2. Distribution of the number of discos/clubs attended by IMD cases likely infected in discos/clubs.

On those days, individuals were sampled to attend a disco/club depending on an age-specific probability obtained from the Italian time-use data (Supplementary Table S2) [S4]. When an individual was selected for disco/club attendance, a venue was assigned to her/him by sampling uniformly from the list of favorite discos/clubs. This ensures that the number of disco/club customers are allocated proportionally to their size. The average attendance to a disco/club was 65% of its capacity, with over-attendance being rare and limited to at most 10% of the disco/club's maximum attendance. In the baseline model, all individuals in a given age group have the same probability to attend a disco/club. This assumption was subject to sensitivity analysis (see Section 3.4).

Age group	Sample size	Probability (%)	Lower 95% CI	Upper 95% CI
16-20	947	12.35	10.33	14.62
21-25	1021	15.18	13.03	17.53
26-30	1191	8.31	6.81	10.03
31-35	1264	3.88	2.88	5.09
36-40	1430	1.33	0.80	2.07

Supplementary Table S2. Probability of attending a disco/club, by age [S4]. The probability of attending a disco/club for age groups below 16 and above 40 years was assumed to be negligible. Sample size is the number of questionnaires compiled by study participants in different age groups on Saturday.

Close contacts

Each individual was assigned a number of close contacts, sampled from a Gaussian distribution with parameters depending on the age group and estimated by contact tracing data from the considered outbreak (Figure1A in the main text and Supplementary Table S3). The average number of contacts was much higher for school-aged children, adolescents and young adults (up to 40 years old) compared to pre-school children (<5 years old) and older adults.

Age group	Mean	Standard deviation
0-4	17.33	5.86
5-40	46.50	17.24
41+	15.00	8.28

Supplementary Table S3. Distribution of the number of contacts by age group (assumed to be Gaussian).

Close contacts assigned to an individual were distributed across the different possible transmission settings. All household members of a given individual were considered close contacts; the remainder was assigned according to age-specific data for Italy from the Polymod contact study [S5] (Supplementary Figure S3). In particular, we considered contacts classified as “work”, “transportation” and “other places” in Polymod as general community contacts in our model, and we sampled them uniformly in the population. Contacts classified as “schools” in Polymod were sampled in the school to which the individual belongs (as random contacts if the individual does not belong to a school); school contacts aged 25+ years represent contacts of university students and schoolteachers or professors. Contacts classified as “leisure” in Polymod were chosen

randomly among individuals sharing the same favorite discos/clubs; if the age of an individual was outside the age-class 16-40 years, they had a negligible probability of attending discos/clubs and therefore leisure contacts were sampled randomly in the population (community contacts).

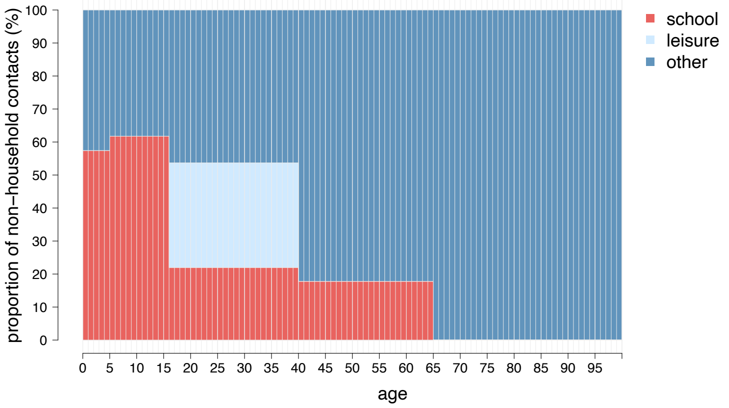

Supplementary Figure S3. Distribution of close contacts outside the household by age of the individual, as used in the model (adapted from [S5]).

Model initialization

We assumed that 0.25% of individuals aged 16-25 years (approx. 800 individuals) are carriers at the beginning of the simulation, using data from [S6] on carriage prevalence in Tuscan provinces not affected by the outbreak. The initial population in the study area was initialized as vaccinated assuming a constant 90.8% coverage at 1 year of age in the last 10 years, 81.9% coverage in individuals aged 11-15 years and 65.9% coverage in individuals aged 15-20 years [S7]. The proportion of effectively protected individuals was adjusted by taking into account the waning of vaccine-induced immunity (exponentially distributed with average 6 years) [S8]. All other individuals were assumed to be susceptible.

Transmission dynamics

Transmission could occur within households (H), schools (S), discos/clubs (D), and in the general community (random transmission R), with rates β_s, where subscript $s \in \{H, S, D, R\}$ indicates the transmission in each of the four considered social settings. At each time step of the simulation (corresponding to 1 day) and for each setting, each susceptible individual has a probability of acquiring carriage equal to $1 - e^{-\beta_s I_s / N_s}$, where I_s and N_s are respectively the number of infectious individuals and total number of individuals in the specific instance of the social setting attended by the susceptible individual (e.g., the household where the susceptible individual lives in, the school attended by the susceptible individual, and so on) [S9-S12]. Susceptible close contacts have an increased probability of becoming carriers according to a relative risk ϕ. A fraction k of individuals who acquire carriage will develop IMD within a few days (with rate ω), and a fraction μ of them will die on the same day of IMD development. Carriers who do not develop IMD will naturally lose carriage with rate γ, or may be decolonized by antibiotic treatment following contact tracing activities.

Antibiotic treatment is assumed to provide protection from carriage for T days. Individuals may be vaccinated after contact tracing or during the immunization campaign, and in such case they will mount a protective immunity after a delay of T_D days, which will wane at rate η. See Supplementary Table S4 for a summary of the adopted values for model parameters and their sources.

PARAMETER	UNIT	VALUE (95%CI)	REFERENCE
RATE OF IMD DEVELOPMENT FOR NEW CARRIERS, $\boldsymbol{\omega}$	$days^{-1}$	0.286	[S13]
CRUDE DEATH RATE AMONG IMD CASES, $\boldsymbol{\mu}$	%	21	Outbreak data
RATE OF NATURAL CLEARANCE OF CARRIAGE, $\boldsymbol{\gamma}$	$days^{-1}$	0.011	[S13-S15]
RELATIVE RISK OF CARRIAGE AMONG CLOSE CONTACTS*, $\boldsymbol{\phi}$	-	2.71	[S16]
TIME TO COMPLETION OF ANTIBIOTIC THERAPY, T	days	10	Outbreak data
TIME TO MOUNTING OF PROTECTION FOR VACCINE*, T_D	days	21	Assumption
WANING RATE FOR VACCINE-INDUCED IMMUNITY, $\boldsymbol{\eta}$	$years^{-1}$	0.166667	[S8]
FRACTION OF INDIVIDUALS ATTENDING THE SAME DISCOS/CLUBS OF AN IMD CASE THAT ARE TRACED*, Z	%	35	Assumption (to comply with outbreak data)
TRANSMISSION PARAMETER IN HOUSEHOLDS, $\boldsymbol{\beta_H}$	$days^{-1}$	6.2 (4.0-8.7) 10^{-3}	Calibration
TRANSMISSION PARAMETER IN SCHOOLS, $\boldsymbol{\beta_S}$	$days^{-1}$	3.9 (1.4-7.4) 10^{-3}	Calibration
TRANSMISSION PARAMETER IN DISCOS/CLUBS, $\boldsymbol{\beta_D}$	$days^{-1}$	0.74 (0.54-0.90)	Calibration
TRANSMISSION PARAMETER IN THE COMMUNITY, $\boldsymbol{\beta_R}$	$days^{-1}$	9.6 (8.2-23.9) 10^{-5}	Calibration
FRACTION OF NEW CARRIERS WHO ACQUIRE IMD, $\boldsymbol{k}$	Cases per 1,000 new carriers	4.6 (1.8-12.2)	Calibration

Supplementary Table S4: model parameters.

** subject to sensitivity analysis (see corresponding section below)*

Supplementary Material S2 – Model calibration

We calibrated the model by using a Markov Chain Monte Carlo framework with a standard recursive-jump Metropolis-Hastings algorithm and uniform priors for the parameters. Given a set of model parameters $\boldsymbol{\theta} = \{\beta_H, \beta_S, \beta_D, \beta_R, k\}$, we defined the model likelihood as the product of the Poisson likelihood of the observed number of IMD cases $C_{s,y}$ in each setting $s = \{H, S, D, R\}$ and year $y = \{2015, 2016\}$ given the corresponding model estimate $\xi_{s,y}$:

$$L(DATA \mid \boldsymbol{\theta}) = \prod_{y}\prod_{s} Pois(C_{s,y}, \xi_{s,y}) \cdot \Theta(\pi_{carriage})$$

where $Pois(k, \lambda)$ is the probability mass function of a Poisson distribution with rate λ, i.e. the probability of observing k events if these events occur with a known rate λ.

The model likelihood was also multiplied by a coefficient Θ to take into account the plausibility of the model-estimated point prevalence of carriage, $\pi_{carriage}$, with respect to results from a prevalence survey conducted in the provinces affected by the outbreak during the period March-June 2016 [S6]. The survey had found 3 carriers in a study population of 1191 individuals aged 11-45 years, enrolled as volunteers among the vaccine recipients of the age-targeted campaign. The estimated point prevalence in this age group was therefore of 2.5 per 1,000 individuals (95% prediction interval, PI: 0.5-7.3). Thus, we computed $\pi_{carriage}$ by replicating the same enrollment scheme in the model and we set Θ to 1 if $\pi_{carriage}$ was within the confidence interval of the observed prevalence, and to 0 if it was outside. In this way, parameter sets producing a prevalence too high or too low were discarded by the Metropolis-Hastings algorithm, while those resulting in plausible prevalence values were accepted or rejected on the basis of estimated $\xi_{s,y}$ values.

Supplementary Material S3 - Scenario analysis

In this section, we assess the effectiveness of the performed interventions by assuming different values of R_e in absence of interventions: in particular, we considered average values of 1.21 and 1.48, corresponding respectively to a decrease and an increase of 10% of the estimated transmission rates ($\boldsymbol{\beta_H}, \boldsymbol{\beta_S}, \boldsymbol{\beta_D}, \boldsymbol{\beta_R}$) with respect to the baseline. Such variations have a disproportionate effect on the estimated number of cases, with on average 43% less cases (95%PI: 14-64%) across 2015-2017 in a scenario with reduced transmissibility, and 84% more cases (95%PI: 22-166%) with enhanced transmissibility (Supplementary Figure S4). Supplementary Figure S5 shows that, even considering the case of R_e = 1.48, the implemented interventions would still be sufficient to push the instantaneous reproduction number, $R_e(t)$, below the epidemic threshold, to a value of 0.84 by the end of 2016, thereby limiting the prevalence of carriage (Supplementary Figure S5). However, the upper 95%PI for $R_e(t)$ at the end of 2016 in this scenario is 0.95, suggesting that containment would likely fail for values of R_e in absence of interventions larger than 1.5.

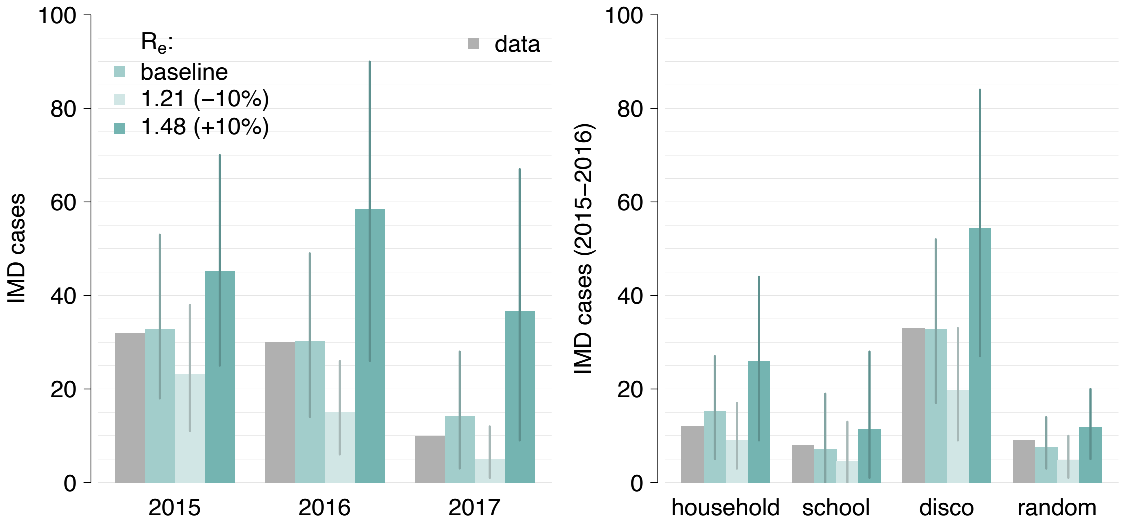

Supplementary Figure S4. Model (with interventions A_c+V_c+V_a) predictions for different values of the effective reproduction number, R_e. Left: average number of IMD cases as predicted by the model (grey bars represent observed data; vertical lines represent 95%PI). Right: average number of IMD cases as predicted the model by setting (Grey bars represent observed data; vertical lines represent 95%PI).

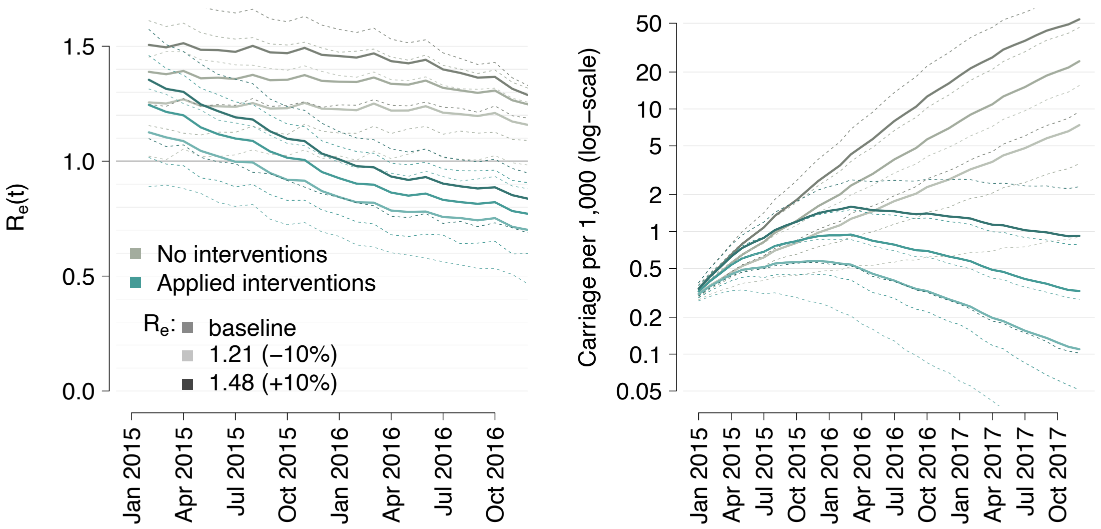

Supplementary Figure S5. Effect of the baseline interventions (A_c+V_c+V_a) for different values of the effective reproduction number, R_e. Left: effective reproduction number over time, with and without interventions (solid lines). Dashed lines represent 95%PI. Right: average carriage prevalence over time, with and without interventions (solid lines, y axis on a log-scale). Dashed lines represent 95%PI.

Supplementary Material S4 - Sensitivity analyses

In this section, we evaluate the effect of changes in specific model assumptions by recalibrating the unknown transmission parameters and risk of IMD development. Recalibration was performed using the same procedure described in Section 1.5. Supplementary Table S5 at the end of this section shows a comparison of model estimates, demonstrating their substantial robustness across different hypothesis.

Delay between vaccination and the mounting of protective immunity, T_D

Here, we evaluate the robustness of the model's assumption on the time required for mounting an effective immune response after immunization. We considered values of 2 or 4 weeks, in addition to the baseline value of 3 weeks, and found substantially no change in any of the model's predictions (see Supplementary Figure S6 and S7), suggesting that uncertainties related to this parameter do not affect much the fundamental transmission dynamics.

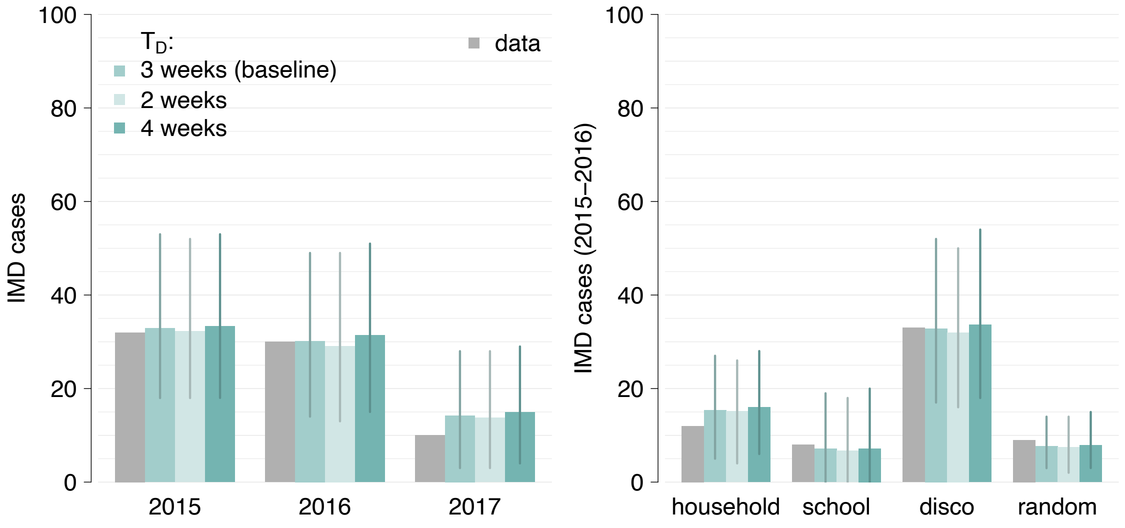

Supplementary Figure S6. Model (with interventions A_c+V_c+V_a) fit and validation by varying the assumed delay between vaccination and the mounting of protective immunity, T_D. Left: observed number of IMD cases by year (grey) over the period 2015-2017 and corresponding average number of IMD cases as predicted by the model (vertical bars represent 95%PI); data for 2017 were not used during calibration and are reported as a model validation. Right: observed number of IMD cases (grey) by setting of acquisition (2015-2016) and corresponding average number of IMD cases as predicted by the model (vertical bars represent 95%PI).

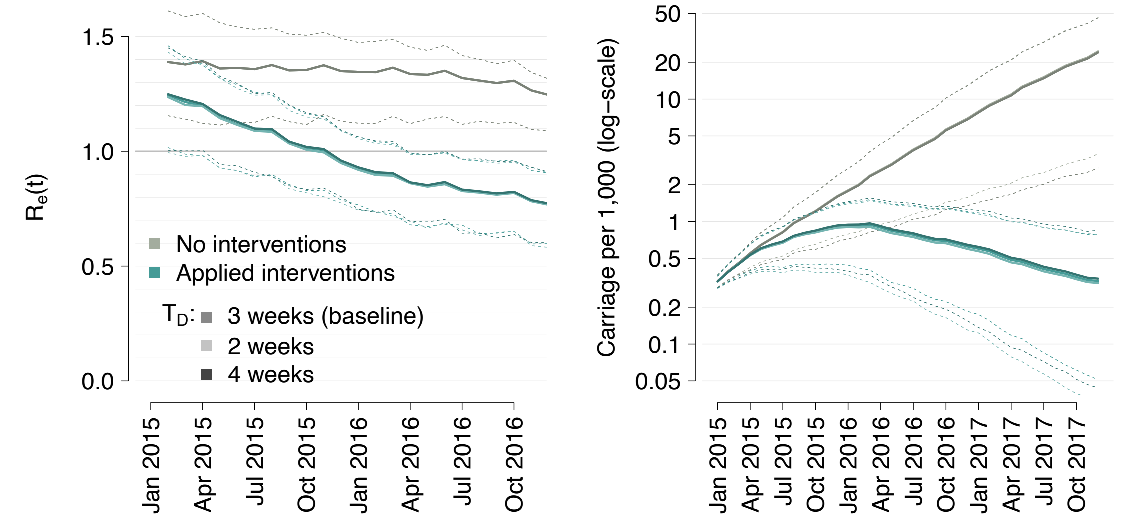

Supplementary Figure S7. Effect of the baseline interventions (A_c+V_c+V_a) by varying the assumed delay between vaccination and the mounting of protective immunity, T_D. Left: effective reproduction number over time, with and without interventions (solid lines). Dashed lines represent 95%PI. Right: average carriage prevalence over time, with and without interventions (solid lines, y axis on a log-scale). Dashed lines represent 95%PI.

Relative risk of carriage among close contacts, ϕ

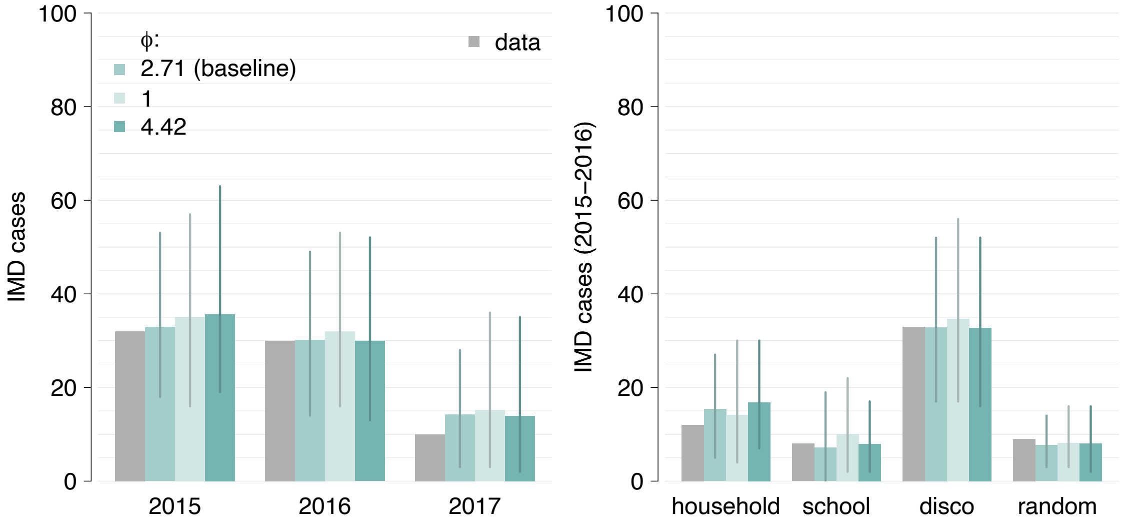

Supplementary Figure S8. Model (with interventions A_c+V_c+V_a) fit and validation by varying the assumed relative risk of carriage among close contacts, ϕ . Left: observed number of IMD cases by year (grey) over the period 2015-2017 and corresponding average number of IMD cases as predicted by the model (vertical bars represent 95%PI); data for 2017 were not used during calibration and are reported as a model validation. Right: observed number of IMD cases (grey) by setting of acquisition (2015-2016) and corresponding average number of IMD cases as predicted by the model (vertical bars represent 95%PI).

In the baseline analysis, we considered close contacts of carriers to be at higher risk of meningococcal acquisition through parameter ϕ, to acknowledge the observation of a carriage prevalence increased by 2.71-fold in close contacts of IMD cases [S16]. Here, we change this assumption in such a way to consider a completely homogeneous transmission within each setting ($\phi = 1$) and, conversely, a scenario where the excess risk of carriage in close contacts is doubled with respect to the baseline ($\phi = 4.42$). The model is equally able to fit the data under these assumptions (Supplementary Figure S8), and the estimated transmission dynamics remain robustly similar to the main analysis (Supplementary Figure S9).

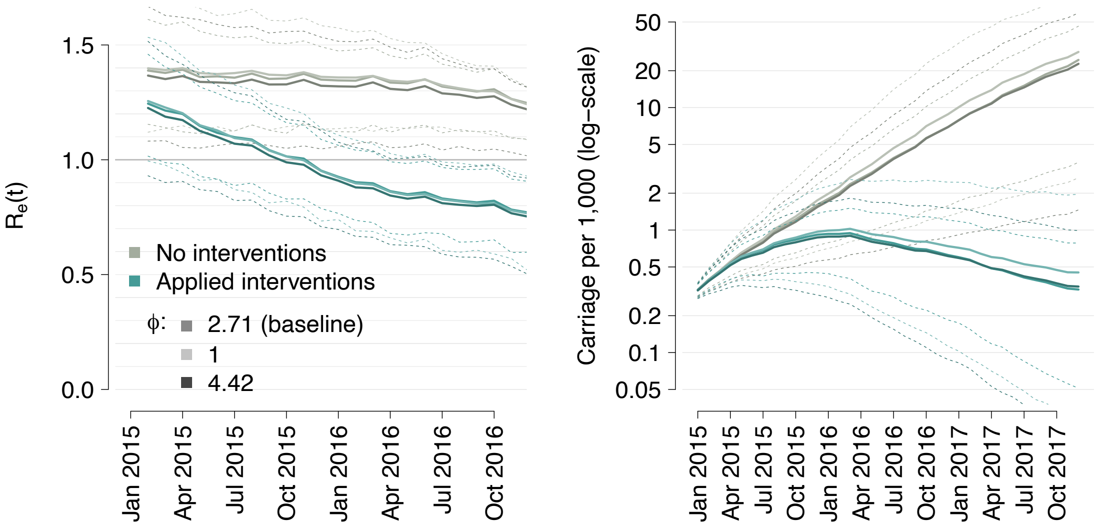

Supplementary Figure S9. Effect of the baseline interventions (A_c+V_c+V_a) by varying the assumed relative risk of carriage among close contacts, ϕ. Left: effective reproduction number over time, with and without interventions (solid lines). Dashed lines represent 95%PI. Right: average carriage prevalence over time, with and without interventions (solid lines, y axis on a log-scale). Dashed lines represent 95%PI.

Fraction of traced disco/club attendees, z

Contact tracing in discos/clubs was one of the main control interventions adopted in the considered outbreak. We assumed that a proportion z of attendees to the same discos/clubs of IMD cases was traced, and in the baseline analysis we fixed this proportion to 35% to comply with the total number of traced contacts. Because this parameter is highly uncertain, we evaluated model predictions under the assumptions of $z = 20\%$ and $z = 50\%$ respectively. Again, the conclusions of the study were substantially unaffected by uncertainties in this parameter (Supplementary Figures S10-S11).

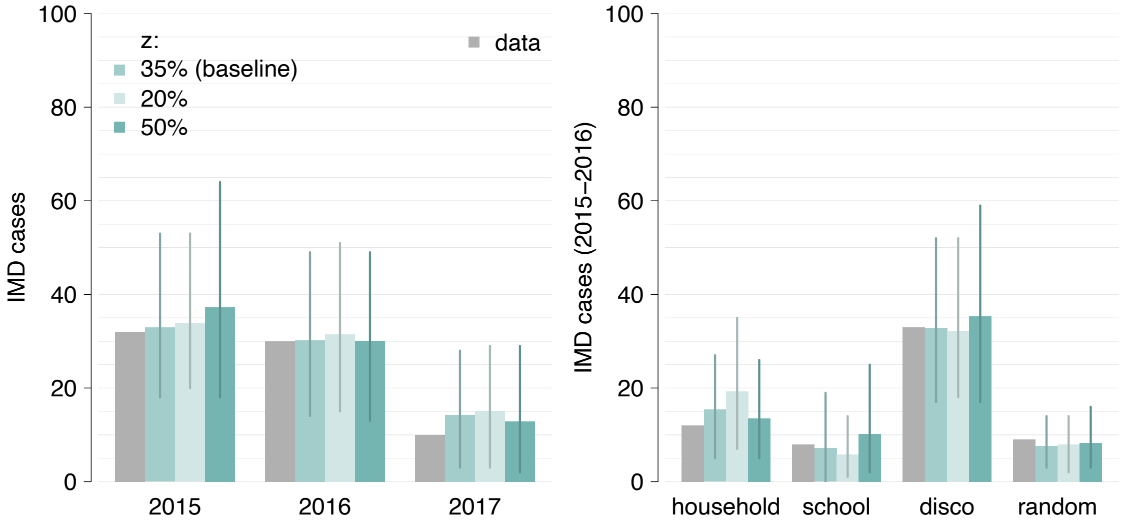

Supplementary Figure S10. Model (with interventions A_c+V_c+V_a) fit and validation by varying the assumed fraction of disco/club attendees that are traced, z . Left: observed number of IMD cases by year (grey) over the period 2015-2017 and corresponding average number of IMD cases as predicted by the model (vertical bars represent 95%PI); data for 2017 were not used during calibration and are reported as a model validation. Right: observed number of IMD cases (grey) by setting of acquisition (2015-2016) and corresponding average number of IMD cases as predicted by the model (vertical bars represent 95%PI).

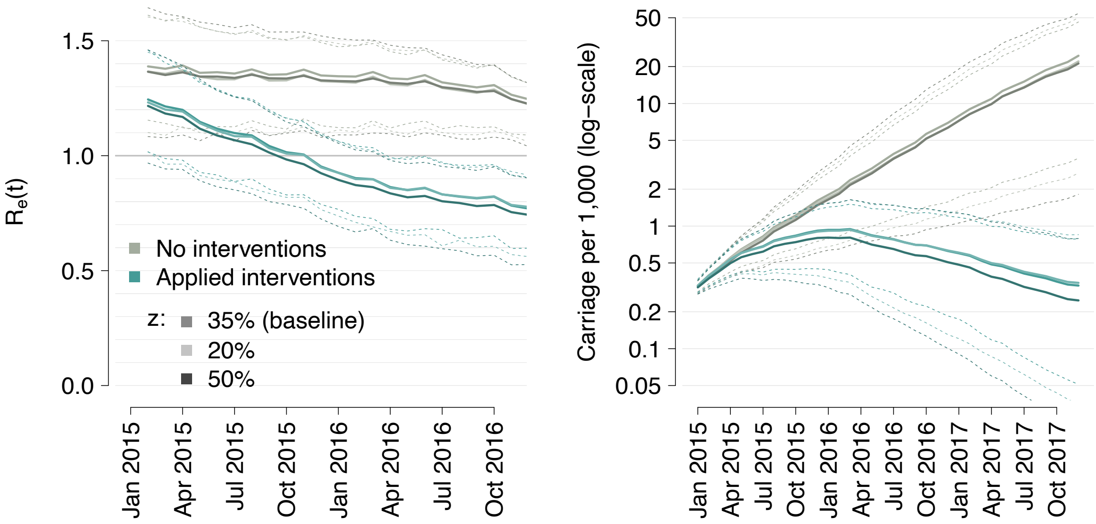

Supplementary Figure S11. Effect of the baseline interventions (A_c+V_c+V_a) by varying the assumed fraction of disco/club attendees that are traced, z. Left: effective reproduction number over time, with and without interventions (solid lines). Dashed lines represent 95%PI. Right: average carriage prevalence over time, with and without interventions (solid lines, y axis on a log-scale). Dashed lines represent 95%PI.

Modeling of disco/club attendees

In the baseline analysis, we assumed for simplicity that all individuals in a given age group are equally likely to attend a disco/club. Here, we consider an opposite assumption, i.e. that some individuals attend discos/clubs every week and all the others never attend discos/clubs (attendance to discos/clubs in practice is probably somewhere in between these two assumptions, with some individuals attending discos/clubs more frequently than others).

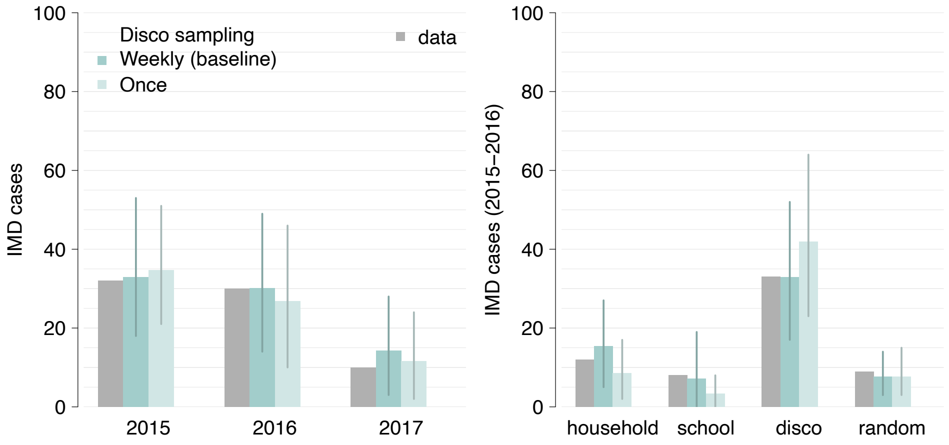

Supplementary Figure S12. Model (with interventions A_c+V_c+V_a) fit and validation by varying the assumed disco/club attendees. Left: observed number of IMD cases by year (grey) over the period 2015-2017 and corresponding average number of IMD cases as predicted by the model (vertical bars represent 95%PI); data for 2017 were not used during calibration and are reported as a model validation. Right: observed number of IMD cases (grey) by setting of acquisition (2015-2016) and corresponding average number of IMD cases as predicted by the model (vertical bars represent 95%PI).

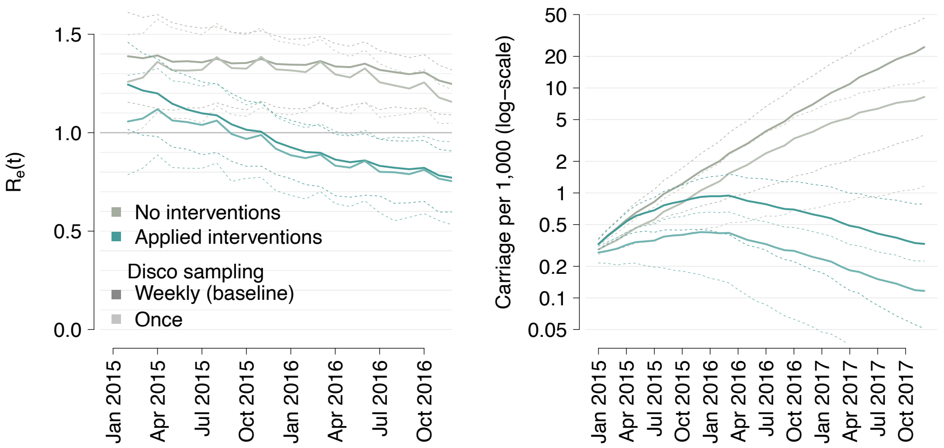

Supplementary Figure S13. Effect of the baseline interventions (A_c+V_c+V_a) by varying the assumed disco/club attendees. Left: effective reproduction number over time, with and without interventions (solid lines). Dashed

lines represent 95%PI. Right: average carriage prevalence over time, with and without interventions (solid lines, y axis on a log-scale). Dashed lines represent 95%PI.

We implemented the latter assumption by modifying the algorithm described in Section 1.1 as follows. Individuals who will attend a disco/club are sampled once and for all at the beginning of each simulation, rather than being re-sampled from the general population every week. Their disco/club of attendance is still re-assigned every week by sampling uniformly from its list of favorite discos/clubs. Supplementary Figure S12 shows that this new model slightly overestimates, on average, the contribution of discos/clubs to IMD cases. Nonetheless, conclusions about the estimated values of the reproductive number remain similar to those of the main analysis, although the prevalence of carriage in this model is assumed to be, on average, slightly lower (Supplementary Figure S13).

Summary of sensitivity analysis results

SCENARIOS		ESTIMATES	
		R_e	k
BASELINE		1.35<br>1.13-1.47	4.6 10^{-3}<br>(1.8-12.2) 10^{-3}
T_D	2 weeks	1.35<br>1.13-1.47	4.6 10^{-3}<br>(1.8-12.2) 10^{-3}
	4 weeks	1.35<br>1.13-1.47	4.6 10^{-3}<br>(1.8-12.2) 10^{-3}
$\boldsymbol{\phi}$	1.0	1.35<br>1.14-1.54	6.0 10^{-3}<br>(1.2-12.9) 10^{-3}
	4.42	1.32<br>1.06-1.50	4.7 10^{-3}<br>(1.8-11.6) 10^{-3}
$\boldsymbol{z}$	20%	1.32<br>1.11-1.47	4.1 10^{-3}<br>(2.0-7.5) 10^{-3}
	50%	1.32<br>1.09-1.49	5.7 10^{-3}<br>(2.1-12.2) 10^{-3}
DISCOS/CLUBS SAMPLING		1.31<br>1.12-1.43	7.5 10^{-3}<br>(4.0-13.7) 10^{-3}

Supplementary Table S5: Estimated reproduction number in the absence of interventions (mean and 95%PI) and estimated fraction of new carriers who acquire IMD (mean and 95%PI) under different hypotheses.

Supplementary References

S1. Fumanelli L, Ajelli M, Manfredi P, Vespignani A, Merler S. Inferring the structure of social contacts from demographic data in the analysis of infectious diseases spread. PLoS computational biology. 2012 Sep;8(9).

S2. ISTAT (2018). Cultura e tempo libero. In: Annuario statistico italiano. [in Italian]

S3. Miglietta A, Fazio C, Neri A, Pezzotti P, Innocenti F, Azzari C, Rossolini GM, Moriondo M, Nieddu F, Iannazzo S, D'ancona F. Interconnected clusters of invasive meningococcal disease due to Neisseria meningitidis serogroup C ST-11 (cc11), involving bisexuals and men who have sex with men, with discos and gay-venues hotspots of transmission, Tuscany, Italy, 2015 to 2016. Eurosurveillance. 2018 Aug 23;23(34).

S4. ISTAT (2003). Indagine multiscopo sulle famiglie. Uso del tempo 2002-2003. [In Italian]

S5. Mossong J, Hens N, Jit M, Beutels P, Auranen K, Mikolajczyk R, Massari M, Salmaso S, Tomba GS, Wallinga J, Heijne J. Social contacts and mixing patterns relevant to the spread of infectious diseases. PLoS medicine. 2008 Mar;5(3).

S6. Miglietta A, Innocenti F, Pezzotti P, Riccobono E, Moriondo M, Pecile P, Nieddu F, Rossolini GM, Azzari C, Balocchini E, Rezza G. Carriage rates and risk factors during an outbreak of invasive meningococcal disease due to Neisseria meningitidis serogroup C ST-11 (cc11) in Tuscany, Italy: a cross-sectional study. BMC infectious diseases. 2019 Dec;19(1):1-7.

S7. Menichetti F, Fortunato S, Ricci A, Salani F, Ripoli A, Tascini C, Fusco FM, Mencarini J, Bartoloni A, Di Pietro M. Invasive Meningococcal Disease due to group C N. meningitidis ST11 (cc11): The Tuscany cluster 2015–2016. Vaccine. 2018 Sep 25;36(40):5962-6.

S8. Campbell H, Andrews N, Borrow R, Trotter C, Miller E. Updated postlicensure surveillance of the meningococcal C conjugate vaccine in England and Wales: effectiveness, validation of serological correlates of protection, and modeling predictions of the duration of herd immunity. Clin. Vaccine Immunol. 2010 May 1;17(5):840-7.

S9. Ciofi Degli Atti M, Merler S, Rizzo C, Ajelli M, Massari M, Manfredi P, Furlanello C, Scalia Tomba G, Iannelli M. Mitigation measures for pandemic influenza in Italy: an individual based model considering different scenarios. 2008, PloS one 3 (3), e1790.

S10. Merler S, Ajelli M, Rizzo C. Age-prioritized use of antivirals during an influenza pandemic. BMC infectious diseases. 2009, 9 (1), 117.

S11. Fumanelli L, Ajelli M, Manfredi P, Vespignani A, Merler S. Inferring the structure of social contacts from demographic data in the analysis of infectious diseases spread. PLoS computational biology. 2012 Sep;8(9).

S12. Fumanelli L, Ajelli M, Merler S, Ferguson NM, Cauchemez S. Model-based comprehensive analysis of school closure policies for mitigating influenza epidemics and pandemics. PLoS computational biology. 2016 Jan 21;12(1):e1004681.

S13. Trotter CL, Gay NJ, Edmunds WJ. The natural history of meningococcal carriage and disease. Epidemiol Infect 2006; 134: 556-566.

S14. Edwards EA, Devine LF, Sengbusch CH, Ward HW. Immunological investigations of meningococcal disease: III. Brevity of group C acquisition prior to disease occurrence. Scandinavian journal of infectious diseases. 1977 Jun 1;9(2):105-10.

S15. Gilmore A, Jones G, Barker M, Soltanpoor N, Stuart JM. Meningococcal disease at the University of Southampton: outbreak investigation. Epidemiol Infect 1999; 123: 185-192.

S16. Kristiansen BE, Tveten Y, Jenkins A. Which contacts of patients with meningococcal disease carry the pathogenic strain of Neisseria meningitidis? A population based study. Bmj. 1998 Sep 5;317(7159):621-5.
